# Beneficial Actions of Essential Fatty Acids in Streptozotocin-Induced Type 1 Diabetes Mellitus

**DOI:** 10.3389/fnut.2022.890277

**Published:** 2022-05-19

**Authors:** Junhui Shen, Li Zhang, Yuanqi Wang, Zhiqing Chen, Jian Ma, Xiaoyun Fang, Undurti N. Das, Ke Yao

**Affiliations:** ^1^Eye Center, Second Affiliated Hospital, School of Medicine, Zhejiang University, Hangzhou, China; ^2^Key Laboratory of Ophthalmology of Zhejiang Province, Hangzhou, China; ^3^UND Life Sciences, Battle Ground, WA, United States; ^4^Department of Biotechnology, Indian Institute of Technology, Kandi, India

**Keywords:** fatty acids, gut microbiome, short-chain fatty acids, bile acids, inflammation, diabetes

## Abstract

The essential fatty acids (EFA), n3 alpha-linolenic acid (ALA), and n6 linoleic acid (LA) are of benefit in diabetes mellitus, but their mechanisms of action are unknown. We, therefore, examined the effects of EFAs on the metabolism, gut microbiota, and inflammatory and retinal histopathology indices in streptozotocin (STZ)-induced type 1 diabetes mellitus (T1DM) animals, and we assessed the levels of vitreal lipoxin A4 (LXA4)—derived from LA—in subjects with diabetic retinopathy (DR). STZ-induced T1DM rats received LA or ALA 100 μg/day intraperitoneally on alternate days for 21 days, and their blood glucose; lipid profile; plasma, hepatic, and retinal fatty acid profiles (by gas chromatography); retinal histology; activities of hepatic and retinal desaturases; and inflammatory markers (by qRT-PCR) were evaluated. Gut microbiota composition was assayed by 16S rDNA sequencing technology of the fecal samples, and their short-chain fatty acids and bile acids were assayed by gas chromatography, liquid chromatography coupled with tandem mass spectrometry, respectively. The human vitreal fatty acid profiles of subjects with proliferative DR and LXA4 levels were measured. LA and ALA significantly improved the plasma glucose and lipid levels; increased the abundance of *Ruminococcaceae* (the ALA-treated group), *Alloprevotella, Prevotellaceae_Ga6A1_group, Ruminococcaceae_UCG_010, and Ruminococcus_1* (the LA-treated group) bacteria; enhanced acetate and butyrate levels; and augmented fecal and hepatic concentrations of cholic acid, chenodeoxycholic acid, and tauro ursodeoxycholic acid in ALA- and LA-treated animals. Significant STZ-induced decreases in plasma LA, gamma-linolenic acid, arachidonic acid, and ALA levels reverted to near normal, following LA and ALA treatments. Significant changes in the expression of desaturases; COX-2, 5-LOX, and 12-LOX enzymes; and cytokines in T1DM were reverted to near normal by EFAs. DR subjects also had low retinal LXA4 levels. The results of the present study show that ALA and LA are of significant benefit in reversing metabolism, gut microbiota, and inflammatory and retinal index changes seen in T1DM, suggesting that EFAs are of benefit in diabetes mellitus.

## Introduction

Type 1 diabetes mellitus (T1DM) is an autoimmune disorder characterized by hyperglycemia and related abnormalities that may lead to the development and progression of various complications, such as neuropathy, nephropathy, retinopathy, cardiomyopathy, and several vascular issues. Despite the availability of effective insulin treatments, long-term consequences, such as diabetic retinopathy (DR), are common in both T1DM and type 2 diabetes mellitus (T2DM). Although there are treatments for DR, including laser photocoagulation, intravitreal injection of anti-vascular endothelial growth factor drugs, and vitrectomy, the exact cause of its onset and progression is not clear. To develop effective therapeutic strategies to prevent T1DM and its complications, an understanding of the pathobiology of the disease is important.

Recent studies have suggested that essential fatty acids (EFA) and their metabolites, such as prostaglandins, lipoxins, resolvins, protectins, and maresins, and changes in gut microbiota—with their ability to influence the synthesis, release, and actions of various cytokines—seem to play critical roles in key physiological functions in the body, including energy metabolism, metabolic signal transmission, and the integrity of the intestinal barrier (1–3). The composition of the intestinal flora can be altered by diet, metabolic diseases, and other factors, and imbalances can activate the innate immune response, promoting chronic inflammation and thus playing an important role in chronic diseases, including diabetes mellitus. A metagenomic analysis of the intestinal flora of 345 Chinese people identified 60,000 molecular markers related to T2DM; it also found that the abundance of butyrate-producing microbes, such as *Clostridiales* sp. *SS3/4*, *Eubacterium rectale*, and *Faecalibacterium prausnitzii*, was lowered, and that the abundance of opportunistic pathogens, such as *Bacteroides caccae, Clostridium hathewayi*, and *Escherichia coli*, was increased (4). Studies have also found that the proportions of bacteria of phylum *Firmicutes* and class *Clostridia* are significantly reduced in diabetic adults, and that the ratios of *Bacteroidetes* to *Firmicutes* and of the *Bacteroides-Prevotella* group to the *C. coccoides-E. rectale* group correlate positively and significantly with plasma glucose concentrations (5). A recent clinical study has compared the intestinal flora of normal people, diabetics without retinopathy, and diabetics with retinopathy and found that the diabetic groups had higher proportions of *Bacteroides*, but that there was no significant difference between subjects with or without retinopathy (6). Yet the mechanism by which gut microbiota can influence the development and progression of DR remains unknown.

The American Diabetes Association recommends replacing saturated fats and trans fats with polyunsaturated fatty acids (PUFA) for those with T2DM in order to modulate the lipid profile (7). α-linoleic acid (ALA) and linoleic acid (LA) are the most abundant omega-3 and omega-6 PUFAs, but their beneficial effects in diabetes mellitus are not clear. In general, omega-3 PUFAs are considered to have anti-inflammatory effects, whereas omega-6 PUFAs are linked to pro-inflammatory events caused by their respective downstream metabolites (8). But some of the metabolites formed from omega-6 LA, such as prostaglandin E1 and lipoxin A4 (LXA4), are potent anti-inflammatory compounds. Hence, linking LA and other omega-6 PUFAs to inflammatory events may not always be true.

A limited number of studies have reported that diets supplemented with n-3 PUFAs modify the diversity of host gut microbiota by increasing the abundance of *Bifidobacterium, Roseburia*, and *Lactobacillus* (9). It has also been reported that long-term intake of LA and ALA may reduce the chances of obese subjects developing diabetes mellitus by modulating gut microbiota (10), and it has further been suggested that PUFAs and their metabolites may play a role in the pathobiology of DR by, at least in part, regulating gut microbiota (11). The influence of gut microbiota on the host’s lipid metabolism may, in turn, be mediated through metabolites produced by the microbiota, such as short-chain fatty acids (SCFA), secondary bile acids (BA), and trimethylamine, and by pro-inflammatory bacterially derived factors, such as lipopolysaccharide (12).

In the current study, we evaluated the effect of LA and ALA on streptozotocin (STZ)-induced T1DM, including their ability to modulate lipid profiles, concentrations of plasma pro-inflammatory cytokines, gut microbiota, SCFAs, BA composition, and DR progression. To determine whether the downstream metabolites of LA/ALA play any role in DR, we also measured vitreal fluid fatty acid profiles and their lipoxin A4 (LXA4) content—the ultimate anti-inflammatory metabolite of LA in those with proliferative diabetic retinopathy (PDR)—because previous studies have shown that LXA4 may play a role in PDR.

## Materials and Methods

### Animals

Male Sprague Dawley rats were obtained from Shanghai SLAC Laboratory Animal Company Limited (Shanghai, China). Animals were housed under barrier-maintained conditions in standard lighting conditions (a 12-h light-dark cycle at 18–25°C) and fed a commercial chow and provided with water *ad libitum*. This study was approved by the animal ethics committee of the Second Affiliated Hospital of Zhejiang University school of medicine. All experimental procedures using the animals were performed in accordance with the ARVO Statement for the Use of Animals in Ophthalmic and Vision Research.

### Induction of Diabetes Mellitus and Fatty Acids Treatment

**FIGURE 1:**
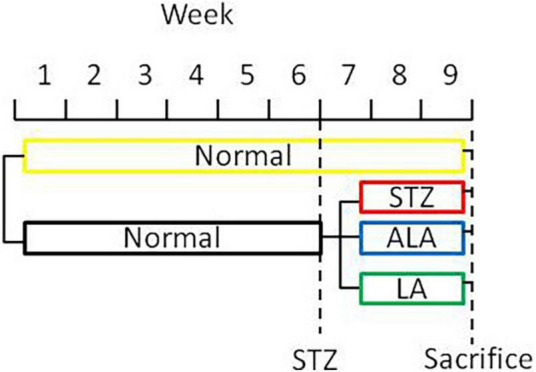
A scheme showing the experimental protocols used in the induction of diabetes and treatment with EFAs (ALA and LA).

Type 1 diabetes mellitus (DM) was induced by STZ (streptozotocin). Male Sprague Dawley rats weighing 200 g that were kept starving for 24 h received a single intraperitoneal injection of 60 mg/kg of freshly prepared STZ (Sigma, United States; Cat No. S0130) in 0.01-M citric acid, pH 4.5. Only those animals showing hyperglycemia (fasting blood sugar ≥ 16.7 mmol/L) for three consecutive days were included in the study. Twenty rats were used in the present study (five rats per group). Only those animals that developed STZ-induced diabetes mellitus received LA or ALA. Thus, we had four groups in the present study: control (no STZ treatment), STZ-induced diabetic animals (that received only STZ), post-ALA-treated diabetic animals (STZ + ALA), and post-LA-treated diabetic animals (STZ + LA), and each group consisted of five animals. The rats in groups STZ + ALA or STZ + LA were treated with ALA (Glpbio Technology Inc., Montclair, CA, United States; Cat No. GC19540) or LA (Sigma, United States; Cat No. L1376) (100 μg per day every other day by intraperitoneal route) after having developed diabetes, respectively, whereas the control and STZ groups were treated with equivalent volume of normal saline. About 21 days after the induction of STZ-induced DM, the rectal feces were collected, and the animals were sacrificed (Figure 1). In a previous study, we showed that AA 10 μg/day per animal given orally or i.p. can prevent both type- 1 and type-2 DMs (13, 14). Since LA is the precursor of AA and ALA that of EPA/DHA and only a fraction of LA and ALA are converted to AA and EPA/DHA (generally, less than 10%) [reviewed in Poorani et al. (15)], we gave LA/ALA 100 μg/animal. The frequency of administration of LA and ALA is also based on our previous studies wherein AA was administered daily for 5 days and subsequently one time a week till the end of the study (30 days). Since the rate of conversion of LA to AA and ALA to EPA/DHA is slow and limited, and hence, to attain reasonable plasma/tissue concentrations of LA and ALA, we administered these fatty acids at the rate of 100 μg/day on alternate days till the end of the study. Furthermore, our previous studies (13, 14) showed that both intraperitoneal and oral administration of AA showed the same degree of beneficial action regarding its ability to prevent chemical-induced diabetes mellitus in experimental animals. This surprising but beneficial action of AA could be attributed to its low molecular weight and ability to get rapidly absorbed from the gut into the systemic circulation. Both LA and ALA are also small molecular weight lipids like AA and, hence, are expected to get absorbed from the gut or peritoneum rather than rapidly like intravenous or intragastric routes of administration. In view of this, in the current study, we administered LA and ALA intraperitoneally.

### Biochemical Assays

Blood was collected from the heart after thoracotomy, and serum was obtained by centrifugation of the clotted blood. Blood (serum) triglyceride (TG), cholesterol (TC), low-density lipoprotein-cholesterol (LDLc) levels, high-density lipoprotein-cholesterol (HDLc), and ApoE levels were measured using an Ortho Clinical Diagnostics VITROS 950 automated analyzer (Johnson & Johnson, New Brunswick, NJ, United States).

### Enzyme-Linked Immunosorbent Assay

The concentration of LXA4 in the vitreous fluid of patients with PDR and controls was measured by human LXA4 ELISA kits (Cusabio, China; Cat No. CSB-E09689h), which followed the manufacturer’s instructions. A microplate reader (Bio-Rad Laboratories iMark microplate reader, United States) was applied to read the absorbance at 450 nm. The minimum detectable concentration by this kit was 0.625 pg/ml.

### Histology

Eyes obtained from the experimental animals were fixed in 10% formalin-glacial acetic acid-ethanol fixative solution (FAA) for 10 min, and then in PBS-buffered 4% paraformaldehyde overnight. The posterior eyecups were dehydrated through a gradient concentration of ethanol from 50 to 100% (each for 30 min), and then in xylene (two times, 10 min each), soaked in paraffin wax (two times, 1 h each). At the end of these treatments, the eyecups were embedded in paraffin blocks (EM-400 Embedding Medium Paraffin; Leica Microsystems, Inc.). The paraffin sections (5 μm) were cut on a paraffin microtome (RM2126RT; Leica Microsystems, Inc.), and stained with hematoxylin and eosin (H&E) for retinal thickness measurement and cell counts.

### Fatty Acids Analysis

To determine the fatty acids composition of the collected plasma and retina samples and vitreous fluid from different groups, they were processed for gas chromatography (GC) as previously described (16). Briefly, for each 100-μl homogenized samples, 1 ml of 0.5 mol/l KOH-methanol solution was added and sealed with nitrogen. After treating these samples in water bath at 60°C for 10 min, 1.5 ml of 14% boron trifluoride methanol solution was added. Then, the samples were transferred to 80°C water bath for 40 min before taking out to cool. About 1.5 ml of n-hexane was added to the samples and vortexed for 1 min. About 2-ml saturated sodium chloride solution was then added before centrifuging at 11,000 rpm for 5 min at room temperature; after which, the samples were left standing at room temperature for 5 min. At the end of this treatment, the supernatant was collected and transferred into clean tubes, and dried under nitrogen at 60°C. To this sample, 1 ml of n-hexane was added to dissolve the extract prior to analysis. An Agilent GC-7890B system (Hewlett Packard Agilent Technologies, California, United States), equipped with a flame ionization detector, was used for fatty acid analysis. The conditions, temperatures of the injection port, and detector were set as previously described (16). A capillary column (DB-23 fused-silica capillary, a 30-m- × −0.25-mm internal diameter, 0.25-μm film thickness; Agilent J&W Scientific, United States) was used for fatty acid analysis.

### Gut Microbiota Analysis

Fresh rectal feces were collected from different groups of animals and frozen in liquid nitrogen till analysis. Gut microbiota was analyzed using 16S rDNA sequencing as described previously (14). Briefly, DNA was extracted from the feces samples using a Micro Elute Genomic DNA Kit (Omega Bio-Tek, Norcross, GA, United States). The V3-V4 region of bacterial 16S rRNA was amplified by using the primers: 338F (5′-ACTCCTACGGGAGGCAGCAG-3′) and 806R (5′-GGACTACHVGGGTWTCTAAT-3′). The PCR product was then extracted from 2% agarose gel and purified using the AxyPrep DNA Gel Extraction Kit (Axygen Biosciences, Union City, CA, United States) according to the manufacturer’s instructions and quantified using Quantus™ Fluorometer (Promega, United States). Purified amplicons were pooled in equimolar and paired-end sequenced on an Illumina MiSeq PE300 platform/NovaSeq PE250 platform (Illumina, San Diego, United States) according to the standard protocols by Majorbio Bio-Pharm Technology Co., Ltd. (Shanghai, China). Sequences were assigned to operational taxonomic units (OTUs) at 97% similarity.

### Determination of Short-Chain Fatty Acids in the Feces

Short-chain fatty acids (SCFAs) concentration in feces was measured by gas chromatography spectrometer (GCS) (7890B Plus GC System, Agilent, California). SCFAs were extracted from stool samples as follows: 100 mg of a dry fecal sample was put in a 10-ml centrifuge tube and gently suspended in 1.6-ml deionized water. About 0.4-ml 50% H_2_SO_4_ and 2-ml diethyl ether were then mixed with an orbital shaker for 45 min before centrifuging at 3,000 rpm for 5 min at room temperature. About 10-mg anhydrous CaCl2 was added to remove residual water for collecting a supernatant. Finally, a 2-μL supernatant was injected into the GCS. Gas chromatography analysis was carried out using Agilent 7890B GCS fitted with a flame ionization detector (FID). A GCS column (ZB-FFAP, Phenomenex, California) of 30 m × 0.32 mm × 0.25 μm was used. Nitrogen was supplied as the carrier gas at a flow rate of 1.69 ml/min in a non-split mode (injector temperature at 250°C). The initial oven temperature was 1006C for 2 min, and then rose at a rate of 8°C/min to 240°C before upholding there for 10 min. The temperatures of the FID and the injection port were 350°C. SCFAs were quantified by an external standard method using the mix standard solution of acetic, propionic, butyric, and valeric acids.

### Determination of Bile Acids

#### Liver Sample Preparation

Frozen liver samples (5–100 mg) were placed in 2-ml tubes containing CK14 ceramic beads (Precellys, Saint Quentin en Yvelines, France). For each 100 mg of tissue, 600 μl of cold methanol and 200 μl of a 1/100 dilution of the IS stock solution were added. Then, liver tissues were homogenized two times for 25 s at 6,000 rpm at 4°C in a Precellys 24 Dual system equipped with a Criolys cooler (Precellys). Tubes were centrifuged at 3,000 g for 5 min at 4°C, and supernatants were transferred into clean tubes. A second BA extraction was performed by 400 μl of cold methanol. Finally, the two extraction supernatants were pooled, aliquoted, and stored at 80°C until the analysis. Aliquots of 150 μl of each homogenate were evaporated to dryness in a Savant speedvac concentrator and later reconstituted in 50 μl of methanol: water (50:50, v/v), centrifuged at 10,000 g for 1 min at 4°C, and transferred into 350-μl volume 96-well plates for further analysis.

#### Stool Sample Preparation

All bile acids (Bas) standards were purchased from Sigma-Aldrich Chemicals Co., and a standard stock solution of each free and conjugated BA was prepared in a concentration of 1 mg/ml in methanol. To be able to make solutions for calibration points, mixture solutions of all free BAs and conjugated BAs were prepared separately at a concentration of 50 μg/ml in methanol. Stool samples, which were kept at -80°C, were allowed to thaw at room temperature, and then accurately weighed 100 mg, added 200 μL (NH4) 2CO_3_. After stood the solution for 5 min samples were spiked with 500 μL internal standard precipitant (4°C) and 500 μL cold methanol. The samples were vortexed for 30 s and extracted for 2 min with ultrasonic technique. Following this, the extract was centrifuged at 13,000 g for 10 min at 4°C, the supernatant was collected and transferred into clean tubes and stored at −20°C. The residue was then reconstituted in 100 μL of Millipore water and transferred into vials to be injected onto the UHPLC-MS/MS system.

Liver and stool samples were analyzed using a Waters Acquity UHPLC system (Waters Corporation, Milford, MA). The samples were injected onto a Waters Acquity UHPLC BEHC18 column (1.8 μm, 2.1 × 100 mm; Waters Corporation). The temperature of the column was 40°C, and the flow rate was 0.4 ml/min. The mobile phases consisted of 10 mmol/L ammonium acetate in water (Eluent A) and 0.1% formic acid in methanol (Eluent B). The gradient elution was performed as follows: 35–60% B (0–3 min), 60–100% B (3–4.5 min). At 4.5 min, the amount of B was kept constant at 100% for 0.5 min and followed by a quick drop to 35% for 1 min and maintained this concentration for 1 min for equilibration and column conditioning. The sample injection volume was 10 μL, and the auto-sampler temperature was set to 4°C. The MS analysis was done using Waters Xevo G2 QTOF (Waters MS Technologies, Manchester, United Kingdom), equipped with an ESI source and operated in the positive ion mode. A capillary voltage of 2,500 V, sample cone voltage of 30 V, a source temperature of 150°C, and a desolation temperature of 450°C were applied. Data were collected in a centroid sensitivity mode in the range of 100–800 m/z, with a lock spray scan collected every 30 s and an average of 3 scans to perform mass correction. For quantification, the m/z of each molecular ion was used with tolerance of 0.03 Da. Individual BAs concentrations, including cholic acid (CA), chenodeoxycholic acid (CDCA), deoxycholic acid (DCA), ursodeoxycholic acid (UDCA), and their corresponding taurine (T)- and glycine (G)-conjugated Bas, were analyzed.

### RNA Extraction, Reverse Transcription, and Real-Time PCR

RNA was extracted from the retina and liver samples with Trizol Reagent (Thermo Fisher Scientific, Invitrogen, United States) as recommended by the manufacturer’s instructions. cDNA was synthesized using PrimeScript™ RT Master Mix (Takara, Dalian, China), using 1 μg of total RNA per reaction. Real-time quantitative PCR was conducted by an Applied Biosystems 7500 Fast Real-Time PCR System using PowerUp SYBR Green Master Mix (Thermo Fisher Scientific, Invitrogen, United States) and 18% of cDNA per reaction. Relative gene expression was determined using the ΔΔCt method by normalizing with the housekeeping gene GAPDH. The sequences for the primers are listed in Table 1.

**TABLE 1:** Primers used for qRT-PCR.

Primer name	Primer sequences (5′–3′)
Rat delta-6-desaturase-forward	AGCTCTCAGATCACCGAGGACTTC
Rat delta-6-desaturase-reverse	ACGATGATGTGGGACAGGAGGAG
Rat delta-5-desaturase-forward	ACGGCATCAAGTATGAGTCCAAGC
Rat delta-5-desaturase-reverse	GTGAAGGTAAGCATCCAGCCAGAG
Rat IL10-forward	CTGCTCTTACTGGCTGGAGTGAAG
Rat IL10-reverse	TGGGTCTGGCTGACTGGGAAG
Rat TNF-α-forward	ATGGGCTCCCTCTCATCAGTTCC
Rat TNF-α-reverse	GCTCCTCCGCTTGGTGGTTTG
Rat IL6-forward	ACTTCCAGCCAGTTGCCTTCTTG
Rat IL6-reverse	TGGTCTGTTGTGGGTGGTATCCTC
Rat GAPDH-forward	ATAGACAAGATGGTGAAG
Rat GAPDH-reverse	TAGAGTCATACTGGAACA
Rat COX-2-forward	AGGTCATCGGTGGAGAGGTGTATC
Rat COX-2-reverse	CGGCACCAGACCAAAGACTTCC
Rat 5-LOX-forward	ACCTATTCCTCCCTGTGCTTCCC
Rat 5-LOX-reverse	CCACACGAGCAGTCCATCATCAC
Rat 12-LOX-forward	CATCTGGTGGCTGAGGTCATTGC
Rat 12-LOX-reverse	GTGTAACGGATGTGCGGAACTAGG

### Patients

This study was approved by the Clinical Ethical Research Committee of the Second Affiliated Hospital of Zhejiang University school of medicine. The study was performed as per the tenets of Helsinki declaration. Informed consent was obtained from all the participating patients. Twenty patients with proliferative diabetic retinopathy (PDR) (10 men and 10 women, average age = 59 years) were recruited for the study. The patients’ diagnosis was based on the international classification standard of DR according to fundus photography and fluorescein angiography. The patients who had a chronic systemic disease (such as hematological or autoimmune disease), hypertension, cancer, high myopia, rhegmatogenous retinal detachment, anti-VEGF therapy or previous vitrectomy surgery, were excluded from the study. Control (*n* = 20, including 10 men and 10 women, mean age = 60 years) samples were obtained from those with idiopathic macula hole who underwent vitrectomy. None of the control patients had diabetes mellitus. Vitreous fluid samples were collected during pars plana vitrectomy (PPV) and reserved at −80°C immediately and centrifuged at 12,500 rpm for 15 min before use.

### Statistical Analysis

Gut microbiota community diversity and richness were analyzed using the R Microbiome Package and R 3.4 software,^1^ including abundance-based coverage estimator (ACE), Chao1 estimator, Shannon, and Simpson indices. Beta diversity was estimated and visualized by principal coordinates analysis (PCoA) plots by QIIME Version 1.8.^2^ Linear discriminant analysis (LDA) effect size (LEfSe, with α = 0.05, Kruskal–Wallis and Wilcoxon tests)^3^ was used to identify significant differences in relative abundances of gut microbiota. The LDA value threshold was set at 3.5. SPSS Version 21.0 (IBM, New York), and Prism Version 6.0 (GraphPad Software) was used for statistical analyses and graph production. The results obtained were expressed as mean ± SEM. Analysis was performed by unpaired two-tailed *t*-test or one-way ANOVA with Tukey’s *post-hoc* test using Prism Version 6.0 (GraphPad Software). The *P*-value of less than 0.05 was considered to be significant.

## Results

### Effects of Alpha-Linolenic Acid and Linoleic Acid on Body Weight, Blood Glucose, and Lipids Status in Streptozotocin-Induced Type 1 Diabetes Mellitus Animals

**FIGURE 2:**
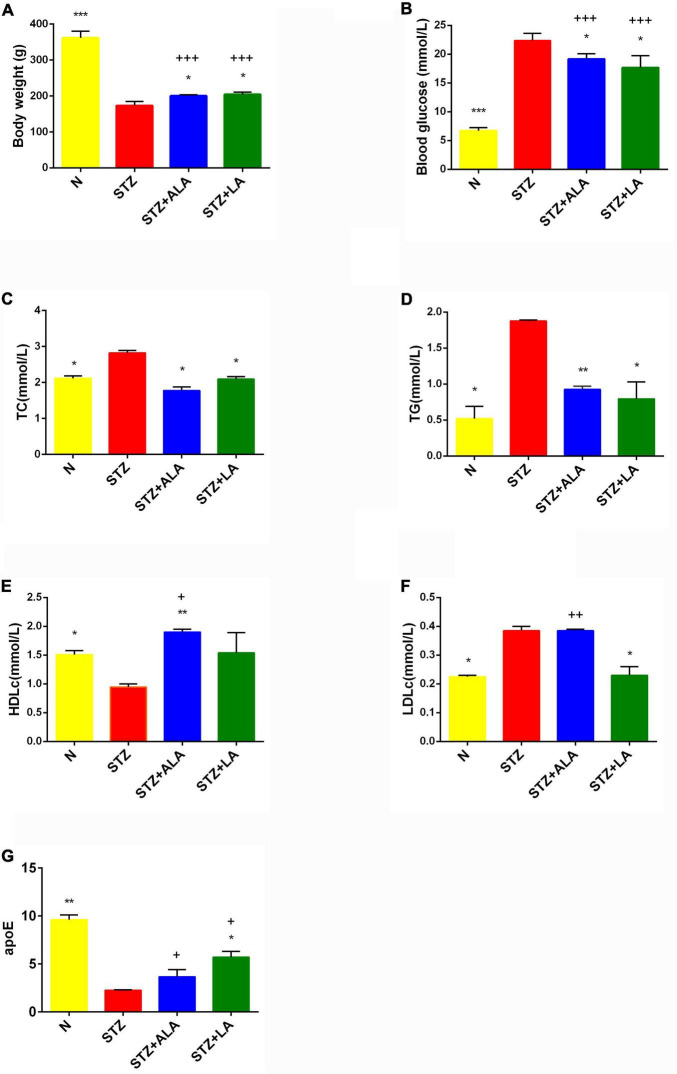
Body weight **(A)**, blood glucose **(B)**, and lipids status **(C–G)** in the plasma of experimental animals. All values are expressed as mean ± SEM (*n* = 5). Comparisons were performed using one-way ANOVA with Tukey’s post hoc test. * versus STZ-treated group. *p ≤ 0.05, **p ≤ 0.01, ***p ≤ 0.001. +*P* ≤ 0.05, ++*P* ≤ 0.01, +++*P* ≤ 0.001.

Following the onset of T1DM, the body weight of the STZ-treated rats decreased 35% in the first week and remained relatively constant between 2 and 4 weeks. In contrast, the body weight of the animals that received ALA and LA along with STZ was considerably lower compared to the group that received STZ, which developed T1DM (Figure 2A). Blood glucose levels in the diabetic group showed a significant 3–4-fold increase compared with the control. In comparison, 3 weeks after the induction of T1DM, those that received LA and ALA showed a significantly smaller increase in blood glucose levels than the T1DM group, implying that LA and ALA can decrease the severity of diabetes induced by STZ (Figure 2B).

STZ-induced T1DM animals showed a significant increase in serum TC, TG, and LDLc and a decrease in HDLc and apoE levels compared with the control. ALA -treated animals (STZ + ALA) showed decreased serum TC and TG and increased HDLc concentrations, while those that received LA (STZ + LA) showed decreased serum TC, TG, and LDLc and increased apoE concentrations compared with the STZ group. Both serum TC and TG levels reverted to near normal in the STZ + ALA and STZ + LA groups, and HDLc and LDLc levels were close to the control values in the STZ + LA group. Thus, both ALA and LA may ameliorate glycolipid homeostasis. The effects of the LA and ALA treatments on glycolipid changes appear slightly different in STZ-induced T1DM animals; LA is superior to ALA in lowering blood glucose, TG, HDL, and LDL, while ALA is better at lowering TG and apoE levels (Figures 2C–G). Thus, supplementation with LA and ALA could be of significant benefit to those with diabetes mellitus for decreasing the severity of hyperglycemia and lipid abnormalities.

### Protection by Alpha-Linolenic Acid and Linoleic Acid of the Diabetic Retina From Reduction in Thickness and Loss of Cells in Different Layers

**FIGURE 3:**
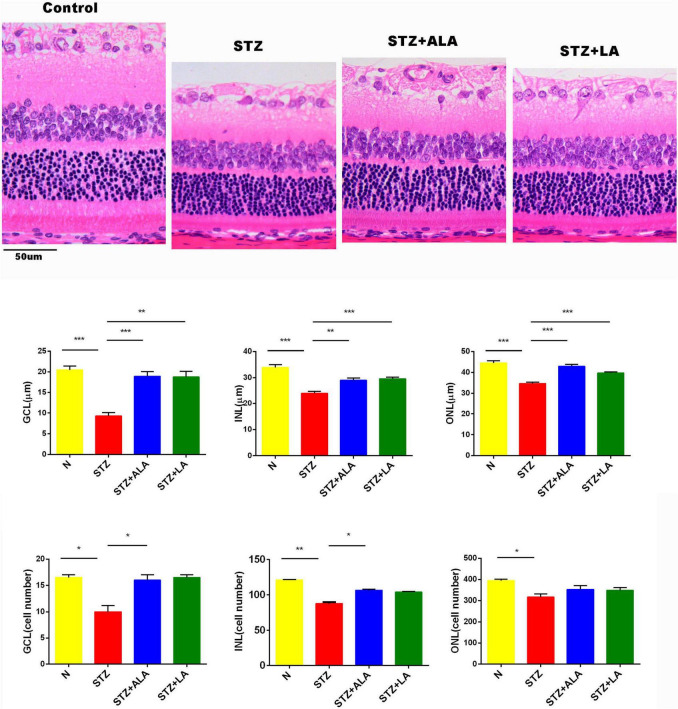
The thickness and the cell number of the retina were measured on HE-stained samples. Magnification, X200. All values are expressed as mean ± SEM (*n* = 5). Comparisons were performed using one-way ANOVA with Tukey’s *post-hoc* test. * vs. the STZ-treated group. *p ≤ 0.05, **p ≤ 0.01, ***p ≤ 0.001.

Morphometric examination of the HE-stained retinal paraffin sections showed significant reductions in the total retinal thickness and the number of cells in the diabetic rats 3 weeks after the onset of T1DM when compared to the non-diabetic control rats (Figure 3). Notably, the reduction of the outer nuclear layer (ONL) was less significant than the changes in the ganglion cell layer (GCL) and the inner nuclear layer (INL). In diabetic rats treated with ALA or LA, the retinal thicknesses of the GCL, INL, and ONL were significantly greater than the STZ group, and the retinal thickness of the GCL had reverted to near that of the control. The number of the cells in the GCL and INL was significantly greater, following ALA or LA treatment, than in the STZ-only group—the number of the cells in the GCL was returned to near that of the control by ALA and by LA treatment, but with no significant difference in the number of the cells in the ONL between the STZ, STZ + ALA, and STZ + LA groups. Based on these results, it can be argued that both ALA and LA prevent or reduce diabetes-induced abnormalities in the retina.

**FIGURE 4:**
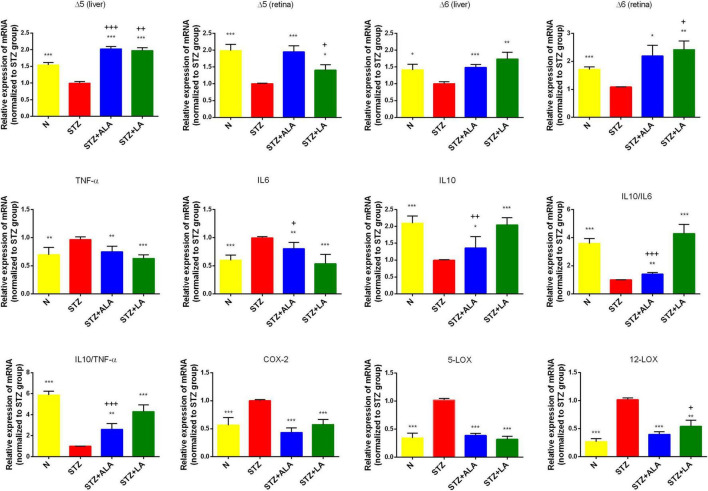
Δ5 desaturase, Δ6 desaturase (in both retina and liver), inflammatory cytokines, COX-2, 5-LOX, and 12-LOX expressions (in the retina only) of the experimental animals. All values are expressed as mean ± SEM (*n* = 5). Comparisons were performed using one-way ANOVA with Tukey’s *post-hoc* test.* vs. the STZ-treated group. *p ≤ 0.05, **p ≤ 0.01, ***p ≤ 0.001. + vs. the normal control group. ^+^*p* ≤ 0.05, ^++^*p* ≤ 0.01, ^+++^*p* ≤ 0.001.

Table 2 presents selected fatty acid profiles in the plasma of the different groups. The differences observed between the normal and STZ groups include a decrease in the values for MUFA oleic acid (18:1 n9), n6PUFA LA (18:2 n6), gamma-linolenic acid (GLA; 18:3 n6), and arachidonic acid (AA; 20:4 n6), as well as an increase in the saturated fatty acid (SFA) palmitic acid (16:0). The fatty acid profile of the STZ + ALA group showed more n3PUFAs α-linolenic acid (18:3 n3) and DHA (22:6 n3) compared to the STZ group, with a concomitant decrease in palmitic acid (16:0). The STZ + LA group showed more n6 PUFAs LA (18:2 n6), GLA, and AA compared to the STZ group. The attainment of plasma LA and AA concentrations close to normal, following LA treatment, suggests that the STZ-induced decrease in Δ^6^ and Δ^5^ desaturases has been reverted to normal. It should be noted that, despite a decrease in GLA levels in the STZ- and STZ + LA-treated groups, the reversion of AA to near normal levels in the STZ + LA-treated group implies that LA treatment can overcome the suppression of desaturases induced by STZ (Figure 4). In contrast to this, STZ treatment resulted in a decrease in ALA levels with no changes in EPA or DHA. The STZ + ALA group showed an increase in ALA and DHA, with no changes in EPA levels. It is rather surprising that STZ-treated animals showed significant decreases in LA and ALA concentrations, which imply that STZ treatment may interfere with the absorption of dietary LA and ALA and/or their incorporation into RBC membranes.

**TABLE 2:** Plasma fatty acid composition of the experimental animals.

Fatty acid composition (%)	Group			
	*N*	STZ	STZ + ALA	STZ + LA
**SFA**				
Palmitic acid (16:0)	22.63 ± 0.81*	28.00 ± 1.24	23.70 ± 0.67*	24.74 ± 0.30
Stearic acid (18:0)	16.55 ± 1.72	13.19 ± 1.16	16.72 ± 0.83	16.21 ± 0.69
**MUFA**				
Palmitoleic acid (16:1n7)	0.47 ± 0.07	0.27 ± 0.05	0.12 ± 0.04	0.17 ± 0.04
Oleic acid (18:1n9)	1.56 ± 0.15*	0.89 ± 0.09	0.69 ± 0.05	0.75 ± 0.04
**n6 PUFA**				
Linoleic acid (18:2n6)	27.43 ± 2.70*	14.05 ± 4.22	21.71 ± 1.73	26.93 ± 0.52*
γlinolenic acid (18:3n6)	0.29 ± 0.06	0.16 ± 0.01	0.16 ± 0.01	0.16 ± 0.02
Arachidonic acid (20:4n6)	17.23 ± 1.71*	11.75 ± 1.08	14.77 ± 1.85	15.95 ± 1.00*
**n3 PUFA**				
αlinolenic acid (18:3n3)	0.26 ± 0.01	0.18 ± 0.03	0.37 ± 0.07*	0.29 ± 0.09
EPA (20:5n3)	0.41 ± 0.03	0.40 ± 0.04	0.40 ± 0.01	0.41 ± 0.08
DHA (22:6n3)	2.57 ± 0.20	2.48 ± 0.34	5.64 ± 0.27 **	2.89 ± 0.17

Data are expressed as mean ± SEM (n = 5). SFA, saturated fatty acid; MUFA, monounsaturated fatty acid; PUFA, polyunsaturated fatty acid; EPA, eicosapentaenoic acid; DHA, docosahexaenoic acid. vs. STZ-treated group, *P ≤ 0.05, **P ≤ 0.01.

Table 3 presents selected fatty acid profiles in the retina. Changes in these profiles were found to be similar to changes observed in the plasma. The retinal fatty acid profile of the STZ-treated group showed decreases in MUFA oleic acid (18:1 n9) and n6PUFA LA (18:2 n6) and increases in palmitic acid (16:0) and stearic acid (18:0). Treatment with ALA resulted in more n3PUFA DHA (22:6 n3), while treatment with LA produced more n6PUFA linoleic acid (18:2 n6) than in the STZ group. It is surprising that LA produced no further decrease in retinal DHA content, while ALA treatments resulted in only a small, although significant, increase in retinal DHA as expected, since ALA is the precursor of DHA (control DHA: 37.64 ± 0.96; STZ: 33.90 ± 1.63; STZ + ALA: 40.35 ± 077; STZ + LA: 32.81 ± 0.97). As expected, the retinal DHA values were significantly different between the STZ + LA and STZ + ALA groups.

**TABLE 3:** Retinal fatty acid composition of the experimental animals.

Fatty acid composition (%)	Group			
	*N*	STZ	STZ + ALA	STZ + LA
**SFA**				
Palmitic acid (16:0)	15.55 ± 0.10**	18.40 ± 0.58	12.97 ± 3.01	14.77 ± 3.77
Stearic acid (18:0)	17.90 ± 0.78*	21.55 ± 0.57	17.25 ± 1.81*	17.95 ± 3.57
**MUFA**				
Palmitoleic acid (16:1n7)	0.23 ± 0.02	0.18 ± 0.01	0.21 ± 0.02	0.16 ± 0.05
Oleic acid (18:1n9)	1.47 ± 0.10*	1.08 ± 0.05	1.20 ± 0.13	0.94 ± 0.34
**n6 PUFA**				
Linoleic acid (18:2n6)	1.54 ± 0.09*	1.07 ± 0.14	1.22 ± 0.24	1.99 ± 0.12**
Arachidonic acid (20:4n6)	8.82 ± 0.44	8.38 ± 0.17	8.69 ± 0.49	10.40 ± 1.58
**n3 PUFA**				
DHA (22:6n3)	37.64 ± 0.96	33.90 ± 1.63	40.35 ± 0.77*	32.81 ± 0.97

Data are expressed as mean ± SEM (n = 5). SFA, saturated fatty acid; MUFA, monounsaturated fatty acid; PUFA, polyunsaturated fatty acid; EPA, ecosapentaenoic acid; DHA, docosahexaenoic acid. vs. STZ-treated group, *P ≤ 0.05, **P ≤ 0.01, ***P ≤ 0.001.

### Alpha-Linolenic Acid and Linoleic Acid Reduced the Severity of Diabetes by Enhancing the Activities of Δ^6^ and Δ^5^ and Suppressed Inflammatory Cytokine Expression

Dietary LA and ALA are converted into their long-chain metabolites—GLA and AA from LA and EPA and DHA from ALA—which form the precursors to various eicosanoids. It is known that the activity of desaturases is decreased in diabetes mellitus, and that this decrease is responsible for low plasma and tissue concentrations of GLA, AA, EPA, and DHA. We, therefore, studied the liver and retina levels of Δ5 and Δ6 desaturases; both of which were significantly lower in the livers and retinas of STZ-treated animals than in the controls (Figures 4A–D) but significantly greater in the ALA- and LA-treated groups than in the STZ group, supporting the contention that the low plasma and tissue levels of GLA, AA, EPA, and DHA in diabetes mellitus (Tables 2, 3 of the present study) are due to low desaturases activity.

Diabetes is considered a low-grade systemic inflammatory condition, and we, therefore, evaluated the mRNA expressions of pro-inflammatory cytokines in the retina. As expected, we observed increased mRNA expressions of IL-6 and TNF-α and decreased IL-10 expression in the STZ-treated animals compared with the control (Figure 4). Retina IL-6, IL-10, and TNF-α levels reverted to near-the-control values in the STZ + LA and STZ + ALA groups. It is notable that there were minor differences in the efficacy of LA and ALA in restoring the retinal desaturases, IL-6, TNF-α, and IL-10 values to near normal. For instance, LA is more effective than ALA in enhancing the activity of Δ6 desaturase in both the retina and the liver, while ALA is superior to LA in enhancing the activity of Δ5 desaturase, also in both the retina and the liver; LA is also superior to ALA in suppressing retinal IL-6 and TNF-α and in enhancing IL-10 expression. In addition, the IL-10/IL-6 and IL-10/TNF-α ratios were greater in the STZ + LA and STZ + ALA groups than in the STZ group, and STZ-induced increases in the expressions of retinal COX-2, 5-LOX, and 12-LOX were restored to near normal by both LA and ALA. Together, these results suggest that both ALA and LA can suppress inflammatory events, although LA seems to be superior to ALA in ameliorating inflammation in the retina.

### Alterations in Gut Microbiota After Essential Fatty Acids Treatment of Streptozotocin-Induced Diabetic Animals

**FIGURE 5:**
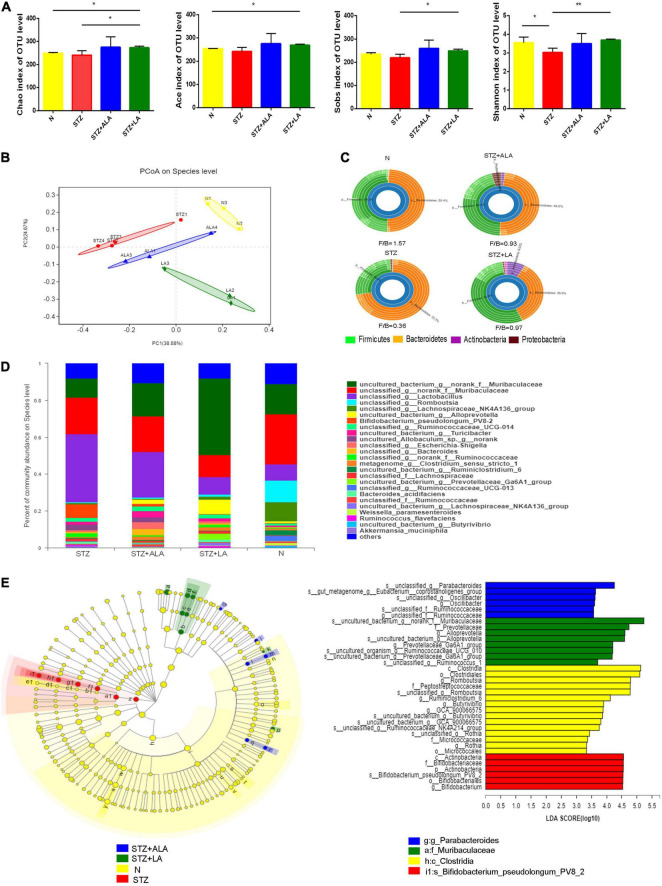
ALA and LA alter gut microbiota composition. Fecal microbiota composition in normal control, the STZ group, the STZ + ALA group, and the STZ + LA group were analyzed using 16S rDNA sequencing (*n* = 3–4). **(A)** Analysis of animals’ feces for the α-diversity of microbiota. Data are expressed as mean ± SEM (*n* = 3–4). *p ≤ 0.05, **p ≤ 0.01. **(B)** Principal co-ordinates analysis (PCoA) based on unweighted UniFrac distance as a measure of β diversity across samples. Each point represents 1 sample. Samples in the same group are labeled the same color. **(C)** Relative abundance of dominant bacteria at the phylum level. **(D)** Relative abundance of dominant bacteria at the genus level. **(E)** LEfSe analysis of enriched bacterial taxa in gut microbiota. LEfSe-derived taxonomic cladogram and the LDA score of enriched bacterial taxa (LDA > 3.5 of LEfSe) were presented. Significantly enriched bacterial taxa in the fecal samples from different groups are indicated by different colors.

Gut microbial α-diversity, such as sobs, chao, and ace, which reflects richness, was compared between the different groups, and we observed that LA treatment resulted in greater microbiota richness than in other groups. The diversity of the observed microbiota species (i.e., Shannon index) was lower in the STZ group than in the N group; however, with LA treatment, the Shannon index increased significantly (Figure 5A). Principal coordinate analysis (PCoA) of the unweighted UniFrac based on the relative species-level abundance showed clustering of the samples according to treatment (Figure 5B). At the phylum level, we observed a dramatic decrease in the *Firmicutes/Bacteroidetes* ratio in diabetic animals compared with normal controls due to both a significant increase in *Bacteroidetes* and a significant reduction in *Firmicutes*, while ALA and LA treatments significantly mitigated this effect (Figure 5C). At the genus taxonomy level, *Bifidobacterium_pseudolonhum_PV8_2* in the STZ group increased significantly compared with the normal controls, whereas the STZ + ALA group saw a large increase in *Ruminococcaceae* compared to the STZ + LA group, which saw increases in *Alloprevotella, Prevotellaceae_Ga6A1_group, Ruminococcaceae_UCG_010*, and *Ruminococcus_1* (Figure 5D). The LEfSe with default parameters was used to identify the key phylotypes, from phylum to genus, responsible for the differences among the four groups studied. LEfSe analysis further illustrated enrichment levels and variations (Figure 5E). Of the differential taxa among the normal control, STZ, STZ + ALA, and STZ + LA cohorts, the normal control group was enriched with genera *Romboutsia*, *Ruminiclostridium_6, Butyrivibrio, GCA_900066575, Ruminococcaceae_NK4A214_group*, and *Rothia*; the STZ group was significantly enriched by phylum *Actinobacteria*, species *Bifidobacterium_pseudolongum_PV8_2*, and genus *Bifidobacterium*; the STZ + ALA group was enriched by genera *Parabacteroides, Eubacterium_ruminantium_group*, and *Oscillibacter*; and the STZ + LA group was significantly enriched by genera *Alloprevotella, Prevotellaceae_Ga6A1_group, Ruminococcaceae_UCG_010*, and *Ruminococcus_1*.

### Alterations of Fecal Short-Chain Fatty Acids After Essential Fatty Acids Treatment in Diabetic Animals

**FIGURE 6:**
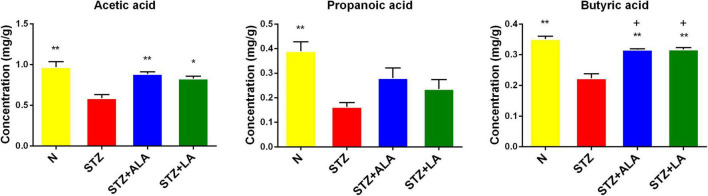
Comparison of fecal SCFA in normal control, the STZ group, the STZ + ALA group, and the STZ + LA group. Data are expressed as mean ± SEM (*n* = 5). *p ≤ 0.05, **p ≤ 0.01. + vs. the normal control group. ^+^*p* ≤ 0.05.

The beneficial actions of gut microbiota have been attributed to their ability to synthesize and release significant amounts of SCFAs, including acetic acid, butyric acid, and propionic acid. We, therefore, measured the fecal concentrations of these using GC. These results showed that the amounts of acetic acid, propionic acid, and butyric acid were significantly decreased following STZ treatment, compared with the control (Figure 6), and that both ALA and LA treatments resulted in significantly greater concentrations of SCFAs than in the STZ group (*p* < 0.01). These results indicate that ALA and LA promote SCFA production, in addition to increasing the abundance of beneficial gut microbiota.

### Essential Fatty Acids Altered Bile Acid Metabolism in Diabetic Animals

**FIGURE 7:**
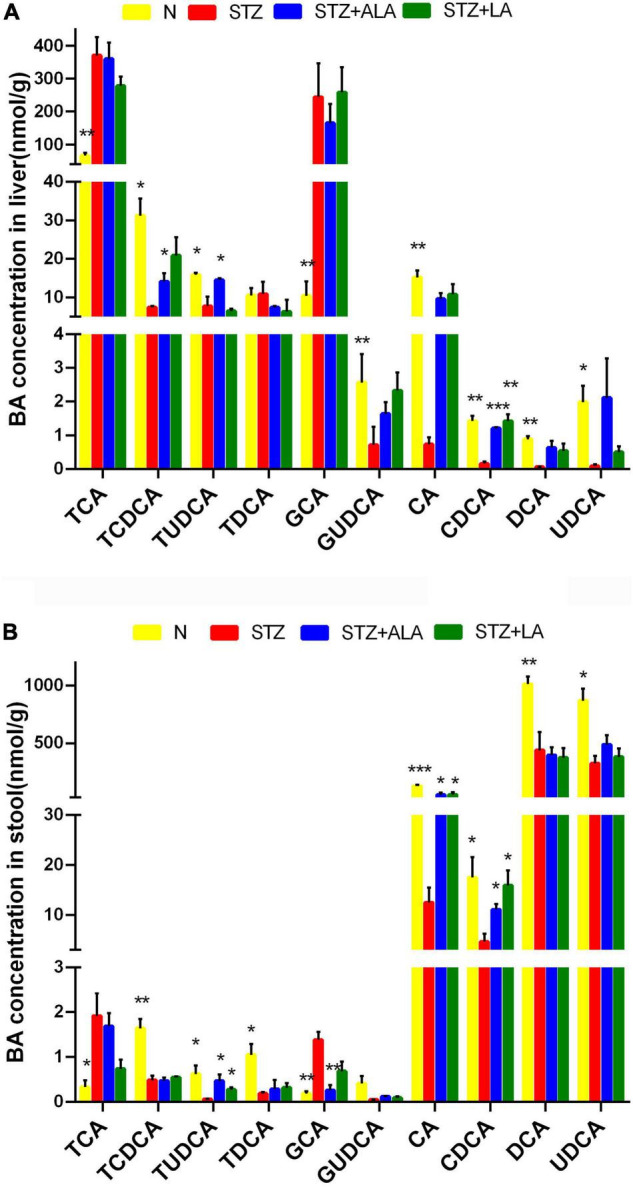
The concentrations of individual BAs in liver tissue **(A)** and stool **(B)**. Results were shown as mean ± SEM (*n* = 5) vs. the STZ-treated group, *p ≤ 0.05, **p ≤ 0.01, ***p ≤ 0.001.

We next tested whether the microbial changes induced by LA and ALA could generate beneficial BA metabolites. BAs are known to alter the gut microbiome composition, and, conversely, the microbiome is known to alter the BA pool. It is evident from the data shown in Figures 7A,B that the STZ group had profoundly lower CA and CDCD concentrations and significantly higher GCA and TCA concentrations than the control group in both the liver tissue and stool. The secondary BAs TUDCA, DCA, and UDCA were also decreased significantly in the STZ group. In the liver tissue, the concentration of primary BAs, such as CDCA and TCDCA, and secondary BAs, like TUDCA, were significantly greater in the ALA group, while the concentration of CDCA was significantly lower in the LA-treated group than in the STZ group. In the stool, both the ALA- and LA-treated groups showed greater CA, CDCA, and TUDCA, while GCA was significantly lower only in the ALA-treated group than in the STZ group.

### Vitreous Fatty Acid Profiles and Levels of Vitreal Lipoxin A4 Levels in the Control and Proliferative Diabetic Retinopathy Groups

**FIGURE 8:**
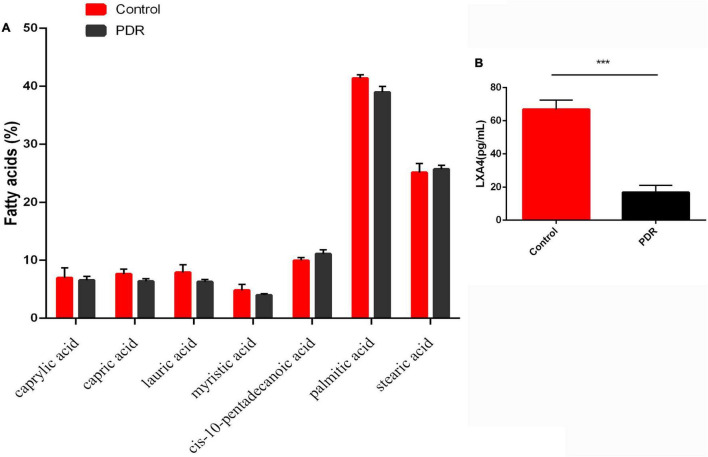
Vitreous fatty acids profile **(A)** and LXA4 levels **(B)** in human control and human PDR groups. All values are expressed as mean ± SEM (*n* = 20). Comparisons were performed using unpaired two-tailed *t* test. ***p ≤ 0.001.

We next investigated whether the changes in the fatty acid profiles observed in the RBC and retina were reflected in the fatty acids present in the human vitreous fluid of the control and the patients with PDR using GC. We could detect only seven fatty acids: caprylic acid, capric acid, lauric acid, myristic acid, cis-10-pentadecanoic acid, palmitic acid, and stearic acid, and there was no significant difference between the PDR group and the control group (Figure 8A). In contrast, LXA4—a potent anti-inflammatory metabolite of AA—was significantly lower in the PDR group than in the macular hole (disease control) group, as shown in Figure 8B.

## Discussion

EFAs LA and ALA are widely available in the human diet, and both are needed for skin integrity, normal immune function, and the integrity of cell membranes. EFA deficiency is known to lead to dehydration caused by loss of skin integrity, immune deficiency, splenic atrophy, and, eventually, death (17–21), although EFA deficiency is currently extremely rare. The importance of both LA and ALA lies in the fact that they form precursors to long-chain metabolites, such as gamma-linolenic acid (18:3, n-6), dihomo-GLA (DGLA; 20:3, n-6), and AA (20:4, n-6)—which are derived from LA—and eicosapentaenoic acid (EPA, 20:5 n-3) and docosahexaenoic acid (DHA, 22:6, n-3)—which are derived from ALA—which are precursors of various eicosanoids that have both pro- and anti-inflammatory actions. LA and ALA and their long-chain metabolites GLA, DGLA, AA, EPA, and DHA—collectively called PUFAs—are important constituents of all cell membranes and thus regulate cell functions and properties and determine cell membrane fluidity (16–21), which, in turn, regulates the expression and affinity of various receptors to their respective substrates. Cell membranes that are more fluid due to high PUAF content thus possess a greater number of insulin receptors and have higher affinity for insulin. This implies that the PUFA content of a cell membrane can influence insulin action and insulin sensitivity (22–29), and PUFAs may, therefore, have an important role in the pathobiology of diabetes mellitus. In addition, EFAs and their metabolites influence the synthesis and action of various cytokines; for instance, it has been shown that AA, EPA, and DHA can suppress the production of the pro-inflammatory cytokines IL-6 and TNF-α (30), and there is evidence to suggest that EFAs and their metabolites influence the production of the anti-inflammatory cytokines IL-4 and IL-10 (30–35). Furthermore, various pro- and anti-inflammatory metabolites formed from EFAs can modulate the functions of macrophages, T cells, NK cells, and CTL cells (36, 37). Thus, EFAs and their metabolites participate in the pathobiology of various inflammatory and autoimmune diseases, including T1DM.

T1DM is an autoimmune disease in which pancreatic β cells are destroyed by infiltrating macrophages and by T cells that secrete IL-6, TNF-α, and interferons. Thus, efforts to suppress these pro-inflammatory cytokines may be of significant benefit in preventing or reducing the severity of T1DM. Since many EFAs and their metabolites can inhibit the production of pro-inflammatory cytokines, it is important to study the effect of these fatty acids in the pathobiology of T1DM. The results of the present study show that both LA and ALA not only decreased the severity of hyperglycemia and altered lipid profiles to near normal but also decreased the expression of retinal pro-inflammatory IL-6 and TNF-α and increased the expression of IL-10, an anti-inflammatory cytokine, to near normal and thus tilted the balance toward anti-inflammation (Figure 4). Furthermore, STZ-induced suppression of the action of delta-6- and delta-5-desaturases in the liver and retina was restored to near normal by LA and ALA (Figure 4). It is notable that STZ induced an increase in the activities of COX-2 and 5-LOX, and that 12-LOX enzymes in the retina were also restored to near normal by LA and ALA. Based on these results, it is expected that, to at least a limited extent, supplementation with ALA and LA could be of benefit in the prevention of DR. This assumption is supported by the observation that ALA- and LA-treated STZ-induced diabetes mellitus rats saw a restoration to near normal of the thickness and number of the cells in the retina (Figure 3). However, we found that there was no significant difference between PDR and macular hole (disease control) conditions with respect to their vitreal fatty acid profiles (Figure 8A), although LXA4—a long-chain metabolite of LA and an anti-inflammatory metabolite derived from AA—was decreased in the human PDR vitreous fluid, indicating abnormal fatty acid metabolism in the PDR (Figure 8B). However, we could not measure vitreal LXA4 levels in the LA- and ALA-treated animals due to the unavailability of animal tissues.

The metabolism of ALA and LA to their respective long-chain metabolites depends on the activities of Δ5 and Δ6 desaturases, which are known to be low in people with diabetes mellitus. This is supported by the results of the present study, which showed that STZ-treated T1DM animals had decreased Δ5 and Δ6 desaturase activity (Figure 4) in the retina and liver tissues, which was restored to normal by ALA and LA supplementation. This suggests that feeding with ALA and LA—the substrates for desaturases—may be of benefit in T1DM. Furthermore, plasma fatty acid composition showed that ALA, LA, AA, EPA, and DHA levels were low (although not to a significant degree) in those with STZ-induced T1DM but reverted to near normal after ALA and LA treatment, attesting to the desaturases also reverting to near normal (Table 2). Similarly, as presented in Table 3, the retinal fatty acid composition showed that decreased LA, AA, and DHA levels induced by STZ treatment could be restored to near normal by LA and ALA treatment (especially DHA levels in ALA-treated rats). These results imply that ALA and LA treatment can restore even retinal fatty acid composition to near normal, which may be responsible for the restoration to near normal of the thickness and the number of the cells of the retina (Figure 3).

AA, EPA, and DHA are the precursors of anti-inflammatory compounds LXA4 (from AA), resolvins of E series (from EPA), and resolvins, protectins, and maresins (from DHA). It is possible that the presence of adequate amounts of AA, EPA, and DHA in the retina is necessary for the formation of LXs, resolvins, protectins, and maresins to prevent DR. In the present study, we could measure only human vitreal fluid LXA4 levels, which were found to be lower in PDR. It remains to be determined whether LXA4, resolvins, and protectins are decreased in animal and human PDR vitreous and, if so, whether they can be restored to normal by ALA and LA treatment. This is supported by the observation that the retina is rich in a variety of PUFAs, whose metabolites play an important role in DR (38–40). We previously showed that ALA could prevent high glucose–induced cellular damage to vascular endothelial cells *in vitro* (41), but it is not known whether ALA’s cytoprotective action is caused by itself or is due to its conversion to EPA and DHA and the subsequent formation of their anti-inflammatory metabolites—resolvins, protectins, and maresins; further studies are needed to determine the process.

The present results extend our previous finding that n3 and n6 PUFAs can prevent STZ-induced diabetes (42, 43). As expected, body weight decreased, blood glucose increased, and dyslipidemia (increased TC, TG, and LDLc, with decreased HDLc and apoE) was observed in the STZ-induced diabetes model, consistent with previous studies (41–43). We found that both ALA- and LA-treated diabetic rats had significantly greater body weight and lower blood glucose. ALA treatment produced lower serum TC and TG and higher HDLc concentrations, while LA treatment produced lower serum TC, TG, and LDLc and higher apoE concentrations, and thus both ALA and LA may ameliorate glycolipid homeostasis. The thicknesses and numbers of the cells of the GCL, INL, and ONL were decreased in STZ-induced diabetes, but these could be restored to near normal by AL2 and LA treatments, which is an encouraging observation suggesting that visual dysfunction due to diabetes mellitus can be prevented, to at least a limited extent, by these fatty acids. Such a therapeutically beneficial action of ALA and LA in DR needs further investigation.

We previously reported that, in alloxan-induced diabetes mellitus rats, the plasma levels of SFAs increased, whereas unsaturated fatty acids decreased (42, 43). Interestingly, in the present study, we found a decrease in MUFA and n6 PUFAs and an increase in SFAs in both the plasma and retinas of STZ-induced T1DM rats (Tables 2, 3). With ALA and LA treatments, most of these abnormalities reverted to normal, except for palmitoleic acid. The present results support the view that supplementation with ALA and LA could be of significant benefit in protecting retinas in DR.

Accumulating evidence suggests that gut microbiota play a major role in the pathobiology of diabetes mellitus. In the present study, we observed that microbial diversity was significantly reduced, following STZ administration, compared to the control. The *Firmicutes/Bacteroidetes* (F/B) ratio has been widely used as an indicator of changes in the microbiome with obesity (44), and, consistent with this (44), at the phylum level, we observed a significant decrease in *Firmicutes* and a significant increase in *Bacteroidetes*, resulting in a dramatic decrease in the *Firmicutes/Bacteroidetes* ratio in the STZ group, while ALA and LA treatment significantly reduced this effect. In addition, at the genus level, increased proportions of lactic acid–producing bacteria, such as *Lactobacillus* and *Bifidobacterium*, in STZ-treated animals have previously been reported (45), which is consistent with our finding that *Bifidobacterium_pseudolonhum_PV8_2* increased significantly in the STZ group. ALA administration reversed this *Ruminococcaceae* increase, consistent with recent work conducted by Zhuang et al. (10). *Ruminococcaceae* are the main microorganisms that convert primary BAs into secondary BAs, and, in the STZ + LA group, *Alloprevotella, Prevotellaceae_Ga6A1_group, Ruminococcaceae_UCG_010*, and *Ruminococcus_1* showed clear increasing trends. Of these, *Alloprevotella* and *Prevotellaceae_Ga6A1_group* are SCFA-producing bacteria (46). *Alloprevotella* is positively correlated with SOD activity and is a genus that ferments carbohydrates and produces acetate and butyrate, which are negatively correlated with various diseases, such as obesity, diabetes, and cardiovascular diseases (47–50). These data, therefore, indicate that ALA and LA administration can result in dramatic restructuring of the microbiota composition of diabetic rats.

Acetic acid, propionic acid, and butyric acid account for 90–95% of total SCFAs (48). Acetate and propionate are the main products of *Bacteroidetes*, and butyrate is mainly produced by *Firmicutes* (49). Butyrate increases insulin sensitivity (50), exerts anti-inflammatory effects (51), regulates energy metabolism, and increases leptin gene expression (52), while n3 PUFA intake is related to the gut microbiota composition and to increasing SCFA production (53). In addition, it has been reported that administrating ALA-rich flax seed oil and safflower oil is beneficial for improving gut microbiota (54). Consistent with these findings (55), the present study showed that the amounts of acetic acid, propionic acid, and butyric acid were decreased significantly in the STZ-induced T1DM group, while ALA and LA administration increased SCFA production, which may account for the observed beneficial actions of EFAs. In addition, increased *Bacteroidetes*, decreased *Firmicutes*, and an increased *Firmicutes-Bacteroidetes* ratio after dietary ALA or LA treatment show that *Bacteroidetes* acted as the main source of the increased acetate.

Intestinal bacteria convert primary BAs into secondary BAs, which are more hydrophilic and less toxic. Studies have shown that TUDCA-producing flora in the intestines of mice protect their retinas by activating the TGR5 pathway (56–60). TUDCA can reduce NO content and downregulate ICAM-1, NOS, NF-κB, and VEGF expressions and thus reduce the development and progression of DR (61). It is likely that UDCA reduces the level of retinal inflammation and attenuates endoplasmic reticulum stress-related retinal pericyte loss and thus arrests DR progression (62–68). Secondary BAs are formed by the gut microbiota, modifying the primary BAs in the large intestinal lumen (67). The feces concentrations of CA, CDCA, and TUDCA were higher and the GCA significantly lower in the groups that received ALA or LA than in the STZ group, and the liver concentrations of CDCA, TCDCA, and TUDCA were significantly higher with ALA treatment and CDCA significantly higher with LA treatment. These results suggest that ALA and LA have favorable effects on glucose homeostasis in STZ-induced T1DM by producing favorable alterations in the intestinal microbiota.

**FIGURE 9:**
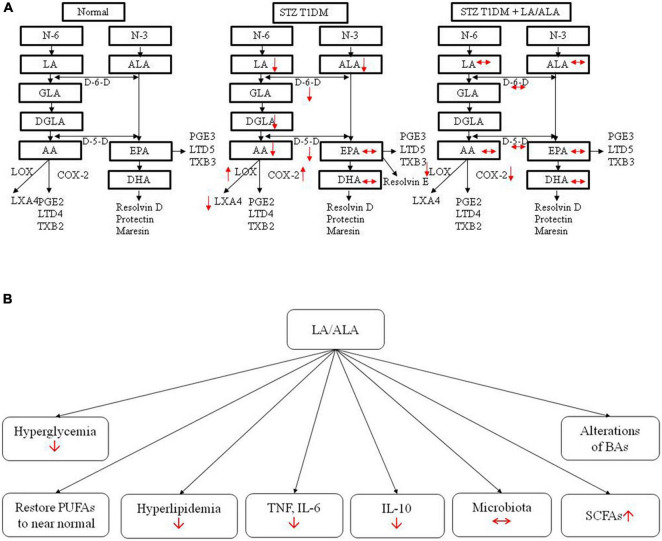
Actions of LA/ALA treatment of STZ-induced T1DM animals **(A)** and potential mechanisms **(B)**. LA, linoleic acid; ALA, alpha-linolenic acid; GLA, Gamma linolenic Acid; DGLA, dihomo-gamma-linolenic acid; PUFA, polyunsaturated fatty acid; AA, arachidonic acid; EPA, eicosapentaenoic acid; DHA, docosahexa.

The results of the present study indicate that ALA and LA treatment can restore many of the abnormalities produced in STZ-induced T1DM animals, including improving diabetic status, diabetes-associated lipid and pro-inflammatory (cytokine) abnormalities, altered gut microbiota, BAs, and retinal structural changes to near normal (Figure 9). Even though LA and ALA administration did change the concentrations of their respective long-chain metabolites, such as GLA, DGLA, and AA (from LA) and EPA and DHA (from ALA), since these changes were not dramatic (see Table 2), it is believed that EFAs themselves may be responsible for the beneficial actions observed. If it is true that EFAs themselves are beneficial in the prevention of development of diabetes and prevent the target organ(s) damage, especially of diabetic retinopathy, relevant clinical studies need to be performed on this account. It remains to be seen whether the beneficial actions of EFAs can also be observed in humans with diabetes mellitus.

## Data Availability Statement

The datasets presented in this study can be found in online repositories. The names of the repository/repositories and accession number(s) can be found in the article/supplementary material.

## Ethics Statement

The studies involving human participants were reviewed and approved by Ethics Committee of the Second Affiliated Hospital, School of Medicine, Zhejiang University. The patients/participants provided their written informed consent to participate in this study. The animal study was reviewed and approved by Ethics Committee of the Second Affiliated Hospital, School of Medicine, Zhejiang University.

## Author Contributions

JS, LZ, UD, and KY conceived and designed the experiments. JS and YW performed the experiments. JS, LZ, YW, and UD analyzed and interpreted the data. LZ, ZC, JM, XF, and KY contributed reagents, materials, and analysis tools. JS and UD drafted the manuscript. JS, LZ, YW, UD, and KY verified the underlying data. All authors reviewed and approved the final version of the manuscript.

## Conflict of Interest

The authors declare that the research was conducted in the absence of any commercial or financial relationships that could be construed as a potential conflict of interest.

## Publisher’s Note

All claims expressed in this article are solely those of the authors and do not necessarily represent those of their affiliated organizations, or those of the publisher, the editors and the reviewers. Any product that may be evaluated in this article, or claim that may be made by its manufacturer, is not guaranteed or endorsed by the publisher.

## Footnotes

1
https://www.r-project.org/

2
http://qiime.org/install/index.html

3
http://huttenhower.sph.harvard.edu/galaxy
